# Apoptosis Enhances the Replication of Human Coronavirus OC43

**DOI:** 10.3390/v13112199

**Published:** 2021-11-01

**Authors:** Sony Maharjan, Mijeong Kang, Jinsoo Kim, Dongbum Kim, Sangkyu Park, Minyoung Kim, Kyeongbin Baek, Younghee Lee, Hyung-Joo Kwon

**Affiliations:** 1 Institute of Medical Science, College of Medicine, Hallym University, Chuncheon 24252, Korea; sonymaharjan3303@gmail.com (S.M.); hahadb@hallym.ac.kr (D.K.); 2 Department of Microbiology, College of Medicine, Hallym University, Chuncheon 24252, Korea; ekf780@naver.com (M.K.); rlawlstn8739@naver.com (J.K.); meany7@naver.com (M.K.); rudqls0905@naver.com (K.B.); 3 Department of Biochemistry, College of Natural Sciences, Chungbuk National University, Cheongju 28644, Korea; pucrim@nate.com

**Keywords:** HCoV-OC43, apoptosis, replication

## Abstract

Human coronavirus OC43 (HCoV-OC43) is one of the coronaviruses causing a mild common cold, but few studies have been made on this strain. Here, we identified the molecular mechanisms involved in HCoV-OC43-induced apoptosis and its implications for viral reproduction in Vero cells and MRC-5 cells. HCoV-OC43 infection induced apoptosis that was accompanied by cleavage of caspase-3 and PARP, degradation of cyclin D1, and cell cycle arrest at S and G2M phases. Dephosphorylation of STAT1 and STAT3, induced by HCoV-OC43 infection, was also associated with HCoV-OC43-mediated apoptosis. The pan-caspase inhibitor effectively prevented HCoV-OC43-induced apoptosis and reduced viral replication, suggesting that apoptosis contributes to viral replication. Collectively our results indicate that HCoV-OC43 induces caspase-dependent apoptosis to promote viral replication in Vero cells and MRC-5 cells.

## 1. Introduction

Coronaviruses (CoVs) are enveloped, positive-sense, single-stranded RNA viruses with a genome of approximately 26–32 kb. Seven strains of human coronaviruses (HCoVs) have been reported to cause mild to severe respiratory infections in humans [1]. The endemic strains HCoV-OC43 and HCoV-229E were first reported in the 1960s, and two new strains, HCoV-NL63 and HCoV-HKU1, were identified in 2004 and 2005, respectively; these HCoVs are known to cause about 15–30% of cases of the common cold [2,3] The first instance of a dangerous CoV as a human pathogen was severe acute respiratory syndrome (SARS) in 2003 with a 9.6% mortality rate [4,5]. In 2012, another HCoV known as Middle East Respiratory Syndrome (MERS) caused an outbreak of a severe respiratory illness with a 37% mortality rate [6]. A novel CoV named SARS-CoV-2 emerged in December 2019 infecting 184 million people worldwide with over 4 million fatalities as of June 2021, establishing it as the worst global pandemic in human history [7].

HCoV-OC43 is a strain that is frequently associated with upper respiratory tract infections and may exacerbate asthma and pneumonia [8,9]. It is occasionally found with other pathologies such as meningitis and enteritis [10,11] and is associated with lower respiratory tract disease and acute disseminated encephalomyelitis in children [12,13,14]. HCoV-OC43 has genes for four structural proteins, spike (*S*), envelope (*E*), membrane (*M*), and nucleocapsid (*N*), and an additional gene for an envelope-associated hemagglutinin esterase (*HE*) protein. Most research has focused on the pathology of SARS-CoV, MERS-CoV, and SARS-CoV-2, while few studies have been conducted on HCoV-OC43. The potential for coronaviruses to cross species barriers with significant mortality risks supports the need for new strategies to prevent and manage HCoV infections.

Apoptosis, or programmed cell death, is a regulated, multistep process that is essential for development, homeostasis, and protection against microbial infection [15]. Coronaviruses and many other viruses stimulate apoptosis during infection [16,17,18,19,20]. Apoptosis is an important antiviral defense of host cells that restricts the spread of infection by killing the virus-infected cells [21,22,23]. HCoV-OC43 induces apoptosis in human lung cells [18] but the underlying mechanisms of this response are not-well known. Here, we characterized apoptosis induced by HCoV-OC43 infection in human lung fibroblast MRC-5 cells along with African green monkey kidney Vero cells. We also examined changes in signaling pathways after HCoV-OC43 infection. Our study may shed light on the mechanisms involved in the pathophysiology of HCoV-OC43 infection.

## 2. Materials and Methods

### 2.1. Cell Culture

Vero cells were obtained from the Korean Cell Line Bank (Product #10081, Seoul, Korea) and MRC-5 cells from the American Type Culture Collection (Product #CCL-171, ATCC, Manassas, VA, USA). Vero cells and MRC-5 cells were cultured in Dulbecco’s modified Eagle’s medium (DMEM, ATCC) and Eagle’s Minimum Essential Medium (EMEM, ATCC), respectively, with 10% fetal bovine serum (FBS, Thermo Fisher Scientific, Waltham, Massachusetts, USA), penicillin (100 U/mL), and streptomycin (100 µg/mL). Cells were maintained in 95% air and 5% CO_2_ at 37 °C.

### 2.2. Virus Amplification

HCoV-OC43 (KBPV-VR-8) was obtained from the Korea Bank for Pathogenic Viruses (College of Medicine, Korea University, Seoul, Korea). HCoV-OC43 at a multiplicity of infection (MOI) of 0.03 was added to 6-well plates of Vero cells (3 × 10^5^ cells/well) after washing with phosphate-buffered saline (PBS) and incubated for 1 h in a 5% CO_2_ incubator at 37 °C with shaking every 15 min. After infection, the plates were maintained with 2 mL of DMEM containing 2% FBS at 37 °C with 5% CO_2_ and harvested at 4 days post-infection_._ The cell culture supernatants were centrifuged for 10 min at 3000 rpm and cleared supernatants were aliquoted and stored at −70 °C. HCoV-OC43 amplification and cell culture were carried out under biosafety level 2 (BSL-2) conditions.

### 2.3. Preparation of Virus-Infected Culture Supernatants

Vero and MRC-5 cells (2 × 10^5^ cells/well) were seeded in 6-well plates in triplicate for each time point and MOI condition. The cells were washed with PBS or DMEM containing 10% FBS, and mock-infected or infected with HCoV-OC43 in PBS or DMEM containing 10% FBS at an indicated MOI in a 5% CO_2_ incubator at 37 °C with shaking every 15 min. After removing the medium, the cells were washed with PBS or DMEM containing 10% FBS. Then, 2 mL of DMEM (for Vero cells) or EMEM (for MRC-5 cells) containing 2% FBS or 10% FBS was added to each well. Cell culture supernatants were harvested at the indicated time points and viral titer was measured using plaque assay and reverse transcriptase-quantitative PCR (RT-qPCR).

### 2.4. Plaque Assay

HCoV-OC43 was quantified by modification of a published plaque assay [24]. Briefly, Vero cells (7 × 10^5^ cells/well) were grown in 6-well plates (Corning, NY, USA) for 18 h. The cells were washed with PBS and infected with ten-fold serial dilutions of the virus-infected culture supernatants for 1 h in a 5% CO_2_ incubator at 37 °C with shaking every 15 min. After removing the medium, 3 mL DMEM/F12 medium (Thermo Fisher Scientific) mixed with 2% Oxoid agar was added to the wells. At 5 days post-infection, the overlay medium was removed, the cells were stained with 0.1% crystal violet, and washed to count plaque formation.

### 2.5. RT-qPCR

Viral RNAs were isolated from virus-infected cell culture supernatants (100 μL) with QIAamp Viral RNA Mini Kit (Catalog No. 52904, Qiagen, Hilden, Germany) as described previously [25]. cDNA (50 μL) was synthesized with a Reverse Transcription System kit (Catalog No. A3500, Promega, Madison, WI, USA). To quantify the nucleocapsid (*N*) gene of HCoV-OC43, the following primers were used (Kim et al., 2020). Forward primer, 5′-CGA TGA GGC TAT TCC GAC TAG GT-3′, reverse primer 5′-CCT TCC TGA GCC TTC AAT ATA GTA ACC-3′, and TaqMan^®^ Probe 5′-FAM-TCC GCC TGG CAC GGT ACT CCC T-TAMRA-3′ [26]. The primers and the probe sequence were synthesized by Genotech (Daejeon, South Korea). GoTaq^®^ Probe qPCR Master Mix (10 µL) (Catalog No. A6101, Promega, Madison, WI, USA) was added to 10 µL reaction mixture containing forward and reverse primers at 125 nM, the probe at 250 nM, and 1 μL cDNA solution. After pre-denaturation at 95 °C for 5 min, 35 cycles of PCR were performed at 95 °C for 15 sec and 60 °C for 1 min using Rotor-Gene Q (Qiagen). The copy number of the *N* gene in the samples was calculated using a standard curve obtained with the TA plasmid vector (Real Biotech Corporation, Banqiao City, Taiwan) containing the HCoV-OC43 *N* gene.

### 2.6. Cell Viability Assay

The viability of HCoV-OC43-infected Vero and MRC-5 cells was assessed by a cell-counting kit-8 (CCK-8) assay (Dojindo Laboratories, Kumamoto, Japan). Briefly, 3 × 10^4^ cells/well were seeded in 24-well plates and cultured for 18 h. The cells were washed with PBS, and mock-infected or infected with HCoV-OC43 in PBS at an MOI of 0.1 or 0.5 for 1 h in a 5% CO_2_ incubator at 37 °C with shaking every 15 min. After removing the medium, the cells were washed with PBS and 2 mL of DMEM (for Vero cells) or EMEM (for MRC-5 cells) containing 2% FBS was added to each well. After 72 h, cell viability was determined by adding CCK-8 reagent and measuring the absorbance at 450 nm following the manufacturer’s instructions.

### 2.7. Apoptosis Assay

Vero and MRC-5 cells (2 × 10^5^ cells/well) were seeded in 6-well plates. The cells were washed with PBS and the cells were mock-infected or infected with HCoV-OC43 in PBS at an MOI of 0.1 and 0.5 for 1 h. After removing the medium, the cells were washed with PBS and 2 mL of DMEM (for Vero cells) or EMEM (for MRC-5 cells) containing 2% FBS was added to each well and cultures for 72 h. After removing the medium, the cells were trypsinized and washed with FACS (fluorescence-activated cell sorting) buffer (1% FBS in PBS) followed by staining with annexin V (eBioscience, San Diego, CA, USA) for 15 min at room temperature in the dark. The cells were further stained with propidium iodide (PI, eBioscience) for 15 min and analyzed with FACSCalibur (BD Biosciences, San Jose, CA, USA). The FACS data were analyzed using Flowing Software (Turku Centre for Biotechnology, Turun Yliopisto, Finland).

### 2.8. Cell Cycle Analysis

For cell cycle analysis, Vero and MRC-5 cells (2 × 10^5^ cells/well) were seeded in 6-well plates. The cells were washed with PBS and then mock-infected or infected with HCoV-OC43 in PBS at an MOI of 0.1 and 0.5 for 1 h. After removing the medium, the cells were washed with PBS and 2 mL of DMEM (for Vero cells) or EMEM (for MRC-5 cells) containing 2% FBS was added to each well and cultures for 72 h. After removing the medium, the trypsinized cells were collected and fixed with 70% ice-cold ethanol in PBS overnight at 4 °C. The cells were subsequently washed with FACS buffer and incubated with RNase (Sigma-Aldrich, St. Louis, MO, USA) for 30 min at 37 °C. Then the cells were stained with PI for 15 min at room temperature in the dark and analyzed with FACSCalibur

### 2.9. Western Blotting

Vero and MRC-5 cells (2 × 10^5^ cells/well) were seeded in 6-well plates. The cells were washed with PBS or DMEM containing 10% FBS, and then mock-infected or infected with HCoV-OC43 in PBS or DMEM containing 10% FBS at an MOI of 0.5 for 1 h. After removing the medium, the cells were washed with PBS and 2 mL of DMEM (for Vero cells) or EMEM (for MRC-5 cells) containing 2% FBS or 10% FBS was added to each well. At various times after infection, culture medium was removed, washed with PBS, and the cells were lysed with lysis buffer (20 mM Tris-HCl pH 8.0, 5 mM EDTA, 150 mM NaCl, 100 mM NaF, 2 mM Na3VO4, 1% NP-40) supplemented with protease inhibitor cocktail tablets (Roche, Basel, Switzerland). The cell lysates were centrifuged at 14,000 rpm at 4 °C for 20 min and equal amounts of proteins were resolved on 4–12% Bis-Tris gradient gels (Thermo Fisher Scientific) and transferred by electrophoresis onto nitrocellulose membranes. Membranes were blocked with 3% bovine serum albumin, incubated with primary antibodies overnight at 4 °C, and then incubated with horseradish peroxidase (HRP)-conjugated secondary antibodies. The protein bands were detected using an enhanced chemiluminescence reagent (Thermo Fisher Scientific) and visualized with ChemiDoc (Bio-Rad, Hercules, California, USA). Antibodies against poly-ADP ribose polymerase (PARP, Catalog No. 9542S), cleaved caspase-3 (Catalog No. 9661S), c-Myc (Catalog No. 5605S), cyclin D1 (Catalog No. 2978S), phospho-STAT1 (Tyr-701) (pSTAT1, Catalog No. 9167S), STAT1 (Catalog No. 14994S), phospho-STAT3 (Tyr-705) (pSTAT3, Catalog No. 9145S), STAT3 (Catalog No. 12640S), phospho-IκBα (pIκBα Catalog No. 9246S) and IκBα (Catalog No. 4814S) were purchased from Cell Signaling Technology (Danvers, MA, USA). Antibodies against HCoV-OC43 N (Catalog No. LS-C79764) and S protein (Catalog No. LS-C371066) were obtained from LifeSpan BioSciences (Seattle, WA, USA). Antibody for β-actin was purchased from Sigma-Aldrich.

### 2.10. Interferon-α Treatment

Vero cells (2 × 10^5^ cells/well) were seeded in 6-well plates. The cells were washed with PBS and then mock-infected or infected with HCoV-OC43 in PBS at an MOI of 0.5 for 1 h. After removing the medium, the cells were washed with PBS, and then 2 mL of DMEM containing 2% FBS and interferon-α-2a (IFN-α-2a, 1000 IU/mL, Sino Biological (Cat. No. 13833-HNAY, Vienna, Austria) was added to each well. At various times after infection, culture medium was harvested and viral titer was measured using plaque assay. The cells were washed with PBS, and the cells were lysed with lysis buffer for western blotting.

### 2.11. Inhibition of Apoptosis

Vero and MRC-5 cells (2 × 10^5^ cells/well) were seeded in 6-well plates and pretreated with Z-VAD-FMK (R&D Systems, Minneapolis, USA) or DMSO for 1 h before HCoV-OC43 infection. The cells were washed with PBS and infected with HCoV-OC43 in PBS at an MOI of 0.1 for 1 h. After infection, Vero and MRC-5 cells were replenished with DMEM containing 2% FBS or EMEM containing 2% FBS, respectively. Supernatants containing viruses from Vero and MRC-5 cells were harvested at 72 h and virus titers were determined by a plaque formation assay. The cell lysates were harvested at 72 h and analyzed by western blot.

### 2.12. Statistical Analysis

Results are shown as the mean ± standard deviation. Differences between the samples were analyzed using an unpaired, two-tailed nonparametric *t*-test of significance (Instat; GraphPad Inc., San Diego, CA, USA). *p*-values < 0.05 were considered statistically significant.

## 3. Results

### 3.1. HCoV-OC43 Replication and Induction of Apoptosis in Vero and MRC-5 Cells

**Figure 1:**
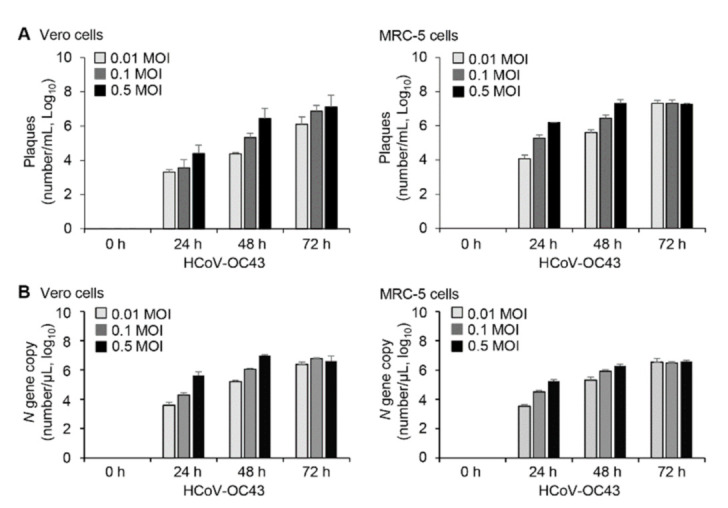
Multiplication of HCoV-OC43 in Vero and MRC-5 cells. Vero and MRC-5 cells (2 × 10^5^ cells/well) were infected with HCoV-OC43 in PBS at an MOI of 0.01, 0.1, or 0.5 for 1 h (*n* = 3). At the indicated times after infection, supernatants were collected and analyzed. (**A**) Virus titers in the supernatants were determined by plaque assays. (**B**) Viral replication was quantified by RT-qPCR analysis for the HCoV-OC43 nucleocapsid (*N*) gene in the supernatants. Copy numbers of the *N* gene in 1 µL of the cDNA samples were calculated using a standard curve obtained with cDNA of the *N* gene.

Viral infection processes can differ depending on cell type; therefore, we used two susceptible cell lines, Vero and MRC-5. Viral infection processes were performed in PBS and then cultured with DMEM (for Vero cells) or EMEM (for MRC-5 cells) containing 2% FBS. To determine the patterns of viral replication and the host response to infection, we used plaque assays (Figure 1A) and quantitative real-time PCR (Figure 1B) to measure HCoV-OC43 in cell culture supernatants harvested at several times after inoculation of cells with HCoV-OC43 at various MOIs. The number of viruses increased in an MOI-dependent and time-dependent manner in Vero and MRC-5 cells (Figure 1). We also performed infection in medium containing 10% FBS and then determined the patterns of viral replication in Vero cells (Figure S1). Compared to the results in 2% FBS condition, virus replication was markedly reduced at 24 h and 48 h by more than 90% irrespective of infected virus MOI in 10% FBS condition. Virus production was similar in both conditions only at 72h after infection with 0.5 MOI of HCoV-OC43. Therefore, we infected and cultured the cells in medium containing 2% FBS for most of the experiments and used 10% FBS condition with an MOI of 0.5 only for control experiments.

**Figure 2:**
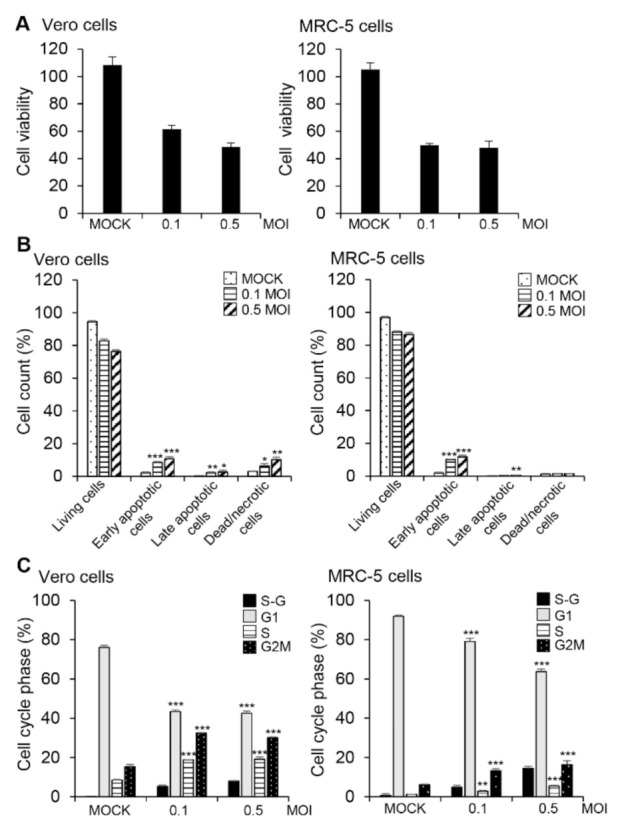
HCoV-OC43 infection triggers apoptosis in Vero and MRC-5 cells. Vero and MRC-5 cells were mock-infected with PBS or infected with HCoV-OC43 in PBS at an MOI of 0.1 or 0.5 for 1 h (*n* = 3). The medium was replaced with DMEM containing 2% FBS or EMEM containing 2% FBS for Vero and MRC-5 cells, respectively. (**A**) Cell viability was determined with CCK-8 assays at 72 h post-infection. (**B**) Cells harvested at 72 h post-infection were stained with annexin V and PI and subjected to FACS analysis. The bar graphs show percentages of cells with indicated properties. (**C**) Cells were stained with PI at 72 h post-infection and the distribution of cells at various phases of the cell cycle was determined by flow cytometry. Each bar graph represents the percentage of cells in each phase of cell cycle. These results are representative of three independent experiments. * *p* < 0.05, ** *p* < 0.01, *** *p* < 0.001.

Since virus-induced apoptosis may benefit viral reproduction [21,27], we assessed the cytotoxic effect of HCoV-OC43 infection in Vero and MRC-5 cells and found that HCoV-OC43 infection at an MOI of 0.1 or 0.5 significantly reduced viability of Vero and MRC-5 cells in comparison to mock-infected control cells (Figure 2A). To determine whether the decrease in cell viability was associated with HCoV-OC43-mediated apoptosis, we measured the exposed phosphatidylserine on the cell membrane using annexin V and PI staining at 72 h post-infection. There was an increase in apoptotic cells after infection with HCoV-OC43 compared to the mock-infection control (Figure 2B and Figure S2A). Vero cells showed higher percentage of dead or necrotic cell population than MRC-5 cells after HCoV-OC43 infection. When we infected the cells with HCoV-OC43 at an MOI of 0.5, 1 or 2 and analyzed at 24 h or 48 h after infection, there was no prominent apoptosis in Vero cells, but necrotic cell population slightly increased after HCoV-OC43 infection compared to mock control (Figure S3A). There was no prominent apoptosis in MRC-5 cells, either (Figure S3B). Therefore, apoptosis seems to occur only at later time points.

Differently from cell viability, most of the analyzed cells were living cells and the apoptotic cell population were comparatively small, only up to a maximum of about 14% (Figure 2B). This can result partly from the difference in assay principles. While cell viability measured with CCK-8 reagent represents enzyme activity of cellular dehydrogenase rather than living cell numbers, readouts of apoptosis assay obtained by FACS represent cell numbers of each population. It is also necessary to consider that the apoptosis assay procedure excludes suspended dead/necrotic cells and debris which are lost during washing steps. There is another possibility that HCoV-43 infection also induces other types of cell death including necroptosis, another form of programmed cell death, in Vero cells and MRC-5 cells as described in neuronal cells [28,29]. As we are interested in STAT signaling and apoptosis, we focused on apoptosis in this study.

Many viruses block cell cycle progression to support viral replication [30]. Therefore, we evaluated the effect of HCoV-OC43 infection on host cell cycle progression and found that a substantial proportion of Vero and MRC-5 cells were found in the S and G_2_/M phases of the cell cycle after infection with HCoV-OC43 (Figure 2C and Figure S2B). Moreover, infection with HCoV-OC43 resulted in an increase of cells at the sub-G_1_ phase in both cell lines even though cell cycle distribution patterns are different in the two cells, further suggesting apoptosis (Figure 2C and Figure S2B). Taken together, these data indicate that HCoV-OC43 infection induced apoptosis and the arrest of cells in the S and G_2_/M phases of the cycle in Vero and MRC-5 cells.

### 3.2. Effects of HCoV-OC43 Infection on Apoptotic and Cell Proliferation Marker Expression

**Figure 3:**
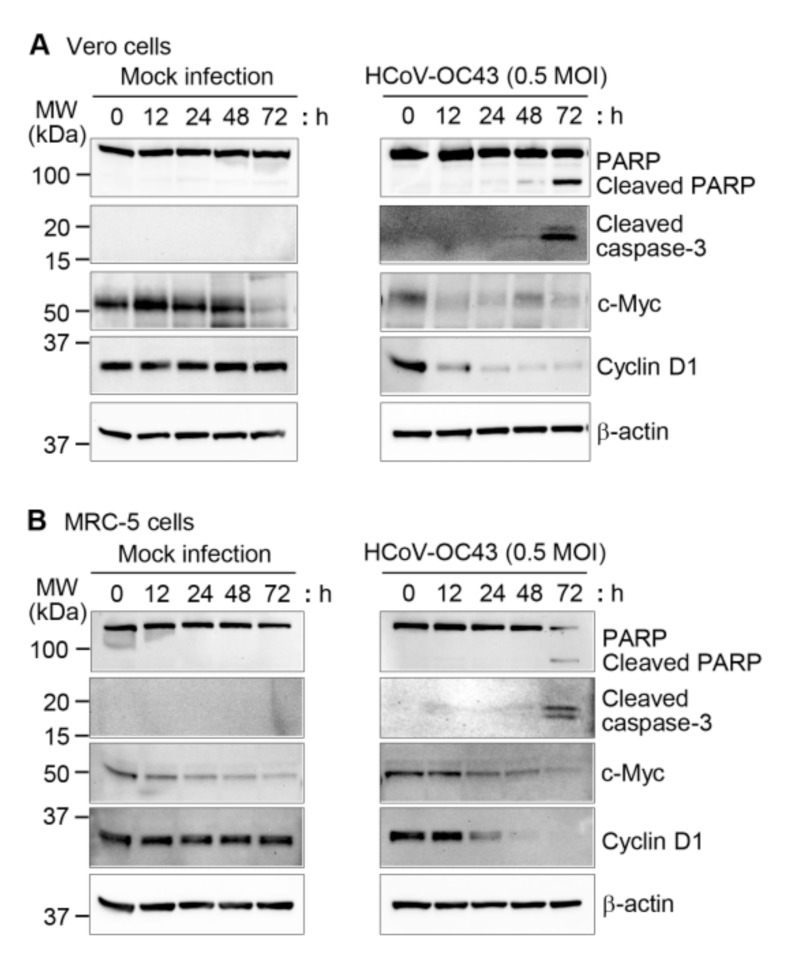
HCoV-OC43 infection affects apoptotic and growth-related marker expression in Vero and MRC-5 cells. Vero (**A**) and MRC-5 (**B**) cells were mock-infected with PBS or with HCoV-OC43 in PBS at an MOI of 0.5 for 1 h. After washing the cells, the medium was replaced with DMEM or EMEM containing 2% FBS for Vero and MRC-5, respectively. After removing the culture medium, cells were harvested at the indicated times and western blots were performed on cell lysates using antibodies to PARP, cleaved caspase-3, c-Myc, and cyclin D1. β-actin antibody was used to verify equal protein loading. The protein band intensities were measured and relative band intensities are shown as a graph in Figure S4. These results are representative of three independent experiments.

Caspases, cysteine-aspartic proteases, are important mediators of apoptosis [31,32], and some viruses use host cell caspases to assist in their replication [33]. PARP is one of the cellular substrates of caspase and cleaved PARP is a hallmark of apoptosis [34]. Therefore, we mock-infected or infected Vero and MRC-5 cells with HCoV-OC43 at an MOI of 0.5 at various time intervals and measured apoptotic and cell proliferation markers using western blots. The amount of cleaved PARP and cleaved caspase-3 increased significantly 72 h after HCoV-OC43 infection in Vero and MRC-5 cells, whereas no differences were observed in the mock-infected cells (Figure 3A,B). We also found that cyclin D1, which is a marker for the proliferation and growth of cells [35], decreased in Vero and MRC-5 cells in response to virus infection compared to mock-infected cells (Figure 3A,B and Figure S4A,B). In contrast, expression of c-Myc, another proliferation marker [36], decreased after HCoV-OC43 infection as well as mock-infection in Vero cells and MRC-5 cells. Therefore, it is likely that the culture condition rather than the virus infection resulted in the decrease of c-Myc (Figure 3A,B and Figure S4A,B). Thus, these data demonstrate that induction of apoptosis was associated with reduced cyclin D1 expression and activation of caspase-3 and PARP in HCoV-OC43-infected Vero and MRC-5 cells.

### 3.3. HCoV-OC43 Infection Decreased Phosphorylation of STAT1 and STAT3 in Vero and MRC-5 Cells

**Figure 4:**
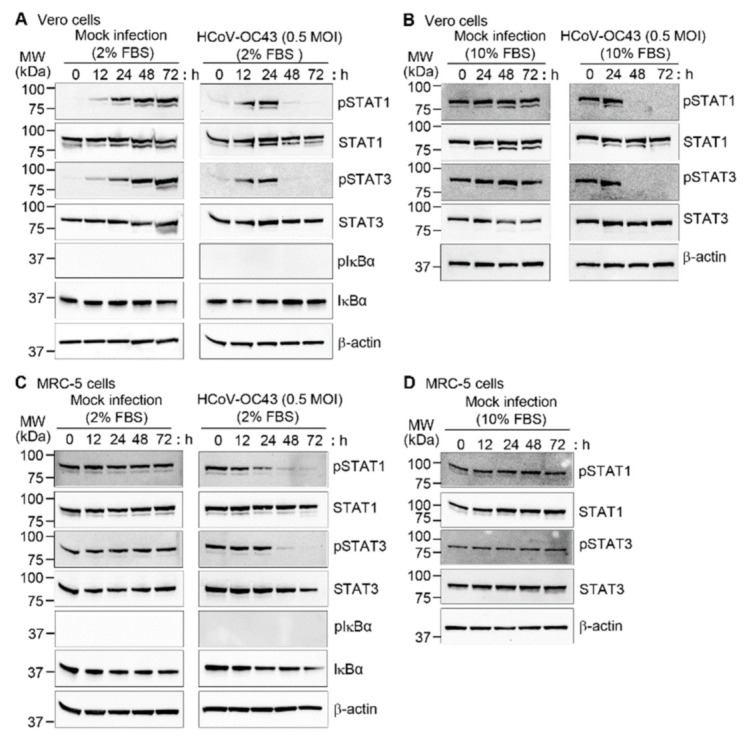
Effect of HCoV-OC43 infection on phosphorylation of STAT1, STAT3, and IκBα. Vero (**A**,**B**) and MRC-5 (**C**,**D**) cells were mock-infected with PBS or infected with HCoV-OC43 in PBS or 10% FBS at an MOI of 0.5 for 1 h. The medium was replenished with DMEM or EMEM containing 2% FBS or 10% FBS for Vero and MRC-5 cells, respectively, and the cells were grown for the indicated times. At the indicated times after infection, cell lysates were examined by western blotting with antibodies against pSTAT1, STAT1, pSTAT3, STAT3, pIκBα, and IκBα. β-actin antibody was used to verify equal protein loading. The protein band intensities of pSTAT1 and pSTAT3 were measured and relative band intensities are shown as a graph in Figure S5. These results are representative of three independent experiments.

Since STAT1 and STAT3 are involved in apoptosis [37,38,39] and viral replication [40,41,42], we measured the expression of phospho-STAT1 and phospho-STAT3 in HCoV-OC43-infected Vero and MRC-5 cells. Vero and MRC-5 cells were mock-infected or HCoV-OC43 infected and harvested at different times. In Vero cells, STAT1 and STAT3 phosphorylation level similarly increased until 24 h culture in medium containing 2% FBS after mock-infection and HCoV-OC43 infection. However, STAT1 and STAT3 phosphorylation decreased sharply by 48 h only after HCoV-OC43 infection (Figure 4A right panel and Figure S5A). In the mock-infected Vero cells, high levels of phosphoproteins persisted until the last time point (Figure 4A left panel and Figure S5A). When we checked phosphorylation levels of STAT1 and STAT3 at 6 h after infection in Vero cells, there was still no prominent effect of HCoV-OC43 infection (Figure S6). It is likely that phosphorylation was induced by the addition of serum-containing medium rather than by virus infection, since mock infection as well as HCoV-OC43 infection was carried out in PBS without serum for 1 h after washing the cells with PBS. To confirm this issue, we performed mock infection in medium containing 10% FBS after washing the cells with the same medium. STAT1 and STAT3 phosphorylation occurred consistently in Vero cells during the mock infection culture as we expected (Figure 4B). When Vero cells were infected with HCoV-OC43 in medium containing 10% FBS, STAT1 and STAT3 phosphorylation decreased sharply by 48 h as was found in 2% FBS condition (Figure 4B right panel). In contrast, the basal phosphorylation levels of STAT1 and STAT3 in MRC-5 cells in 2% FBS condition were high, and the addition of 10% FBS containing medium did not affect phosphorylation (Figure 4C,D and Figure S5B). Considering that Vero cells are defective in the type I interferon genes [43], IFN signaling might be related to the higher STAT phosphorylation levels in MRC-5 cells. However, HCoV-OC43 infection reduced the phosphorylation levels at later time points, similar to Vero cells (Figure 4C right panel and Figure S5B). Because the NF-κB signaling pathway is activated by several viruses, we measured IκBα phosphorylation and degradation, but no significant changes were observed in Vero and MRC-5 cells with or without HCoV-OC43 infection. These results indicate that HCoV-OC43 infection specifically reduced STAT1 and STAT3 phosphorylation, which could induce apoptosis in the infected cells.

### 3.4. Suppression of Apoptosis and Reduction of the Viral Load by Caspase Inhibition

**Figure 5:**
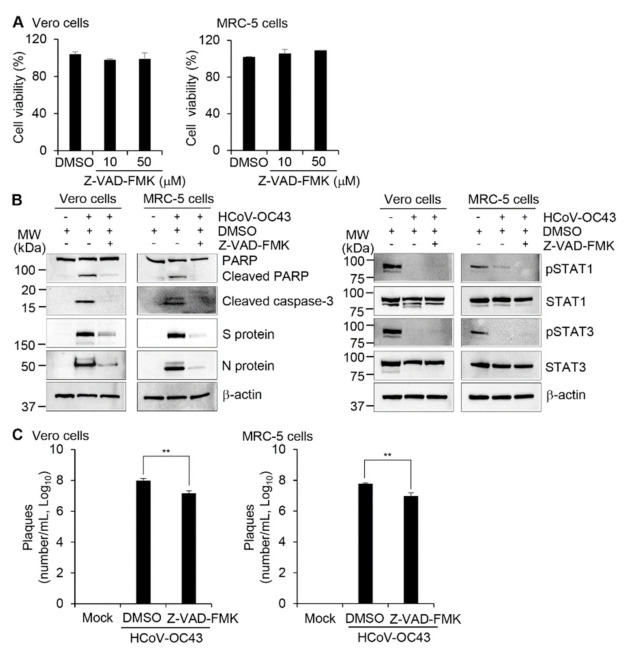
Treatment with a caspase inhibitor attenuates HCoV-OC43 replication. (**A**) Vero and MRC-5 cells were treated with DMSO or the indicated concentration of Z-VAD-FMK for 72 h. Cell viability was determined by CCK-8 assays. (**B,C**) Vero and MRC-5 cells were treated with DMSO or Z-VAD-FMK (50 µM) for 1 h, followed by mock infection or HCoV-OC43 infection at an MOI of 0.1. (**B**) The lysates of cells harvested 72 h after infection were examined by western blotting using antibodies against PARP, cleaved caspase-3, and HCoV-OC43 N and S proteins (left) and pSTAT1, STAT1, pSTAT3, STAT3 (right). β-actin antibody was used to verify equal protein loading. (**C**) Virus titers from supernatants collected at 72 h post-infection were determined by plaque assays. ** *p* < 0.01.

To determine whether the apoptotic changes in HCoV-OC43-infected cells were caspase-dependent, we used z-VAD-FMK, an inhibitor of various caspases, including caspase-3 [44]. Vero and MRC-5 cells treated with z-VAD-FMK at 10 µM and 50 µM and then assayed using CCK-8 showed no evidence of cytotoxicity (Figure 5A). To determine whether z-VAD-FMK could inhibit HCoV-OC43-induced apoptosis in Vero and MRC-5 cells, we measured the cleavage of caspase-3 and PARP protein. Treatment of HCoV-OC43-infected Vero and MRC-5 cells with z-VAD-FMK reduced the amount of cleaved caspase-3 and blocked cleavage of PARP (Figure 5B left panel). To measure virus production, the expression of HCoV-OC43 N and S proteins was determined in the presence and absence of z-VAD-FMK by western blot analysis. z-VAD-FMK significantly reduced the levels of viral N and S proteins in the virus-infected cells (Figure 5B left panel). However, z-VAD-FMK did not affect STAT1 and STAT3 phosphorylation status suggesting that apoptosis can be a downstream event of STAT dephosphorylation (Figure 5B right panel). Plaque formation assay showed that HCoV-OC43 replication was reduced in the presence of z-VAD-FMK in Vero cells and MRC-5 cells (Figure 5C) by about 1 log. More specifically, virus titer was reduced by 81.9% for Vero cells and 82.3% for MRC-5 cells in the presence of z-VAD-FMK. These results suggest that HCoV-OC43-induced apoptosis contributes to virus replication in Vero and MRC-5 cells.

### 3.5. Activation of STAT 1 and STAT3 and Reduction of the Viral Load by Interferon-α

**Figure 6:**
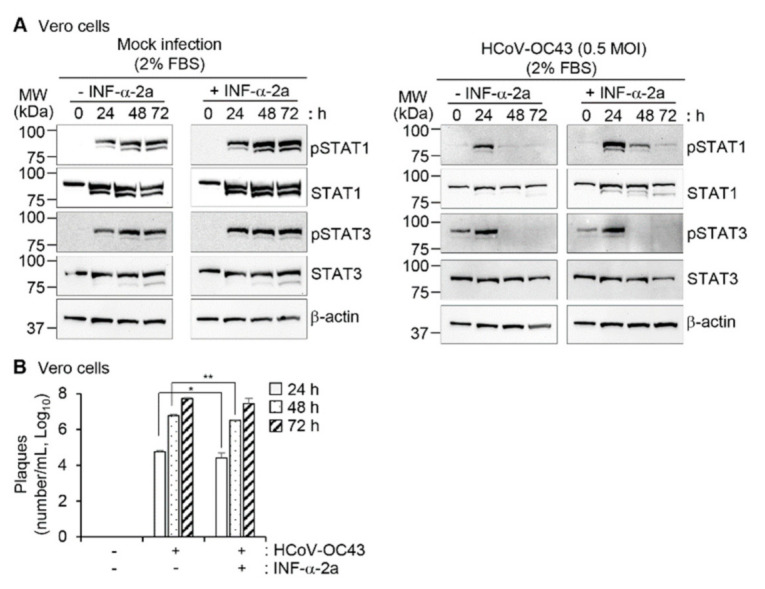
Effect of IFN-α-2a on STAT1 and STAT3 phosphorylation and viral replication. (**A**) Vero cells were mock-infected with PBS or infected with HCoV-OC43 in PBS at an MOI of 0.5 for 1 h. The medium was replenished with DMEM containing 2% FBS with or without 1000 IU/mL of IFN-α-2a. At the indicated times after infection, cell lysates were examined by western blotting with antibodies against pSTAT1, STAT1, pSTAT3, and STAT3. β-actin antibody was used to verify equal protein loading. The protein band intensities of pSTAT1 and pSTAT3 were measured and relative band intensities are shown as a graph in Figure S7. (**B**) Virus titers from the cell culture supernatants collected at the indicated times after infection were determined by plaque assays. * *p* < 0.05, ** *p* < 0.01.

Interferon-α-2a (IFN-α-2a) is one of type I interferons which are known to induce activation of STATs including STAT1 and STAT3 [45]. As we found that STAT1 and STAT3 dephosphorylation is associated with apoptosis and apoptosis contributes to virus replication, we investigated the effects of IFN-α-2a after HCoV-OC43 infection in Vero cells (Figure 6A and Figure S7). Treatment with 1000 IU/mL of IFN-α-2a increased STAT1 and STAT3 phosphorylation in mock-infected cells as we expected (Figure 6A left panel). IFN-α-2a clearly delayed reduction of STAT1 phosphorylation and slightly suppressed STAT3 phosphorylation in response to HCoV-OC43 infection at an MOI of 0.5 (Figure 6A right panel and Figure S7). Accordingly, virus production was reduced in the presence of IFN-α-2a by 54.3% (0.34 log decrease) and 46.3% (0.27 log decrease) at 24 h and 48 h after infection, respectively, compared to the results in the absence of IFN-α-2a. Considering that we used 0.5 MOI of HCoV-OC43, this outcome is consistent with the previous report regarding the effect of IFN-α-2b on SARS-CoV replication in Vero E6 cells; treatment with 1000 IU/mL of IFN-α-2b reduced about 0.5 log decrease in virus titers at 72 h after infection with 0.001 MOI of SARS-CoV [46]. These results suggest that IFN-α-2a-induced increase of STAT1 and STAT3 phosphorylation contributes to the suppressive effect of IFN-α-2a on virus replication in Vero cells.

## 4. Discussion

Programmed cell death or apoptosis is an essential host cell response to viral infection [47,48]. In order to survive and propagate, many viruses have evolved methods to inhibit apoptosis and promote their replication [49,50]. In contrast, other viruses take advantage of host cell apoptosis to escape the host immune system. Moreover, apoptosis of infected cells facilitates the release of progeny viruses and their transmission to other cells. This response is also important for the pathogenesis of the virus, leading to cell death and tissue damage [27,48]. Several coronaviruses such as swine acute diarrhea syndrome coronavirus (SADS-CoV), porcine epidemic diarrhea virus (PEDV), porcine delta coronavirus (PDCoV), avian infectious bronchitis virus (IBV), and mouse hepatitis virus (MHV) have various strategies to modulate apoptotic pathways in different cell lines to promote viral replication and propagation [51]. However, little information is available on HCoV-OC43-induced apoptosis or the underlying cellular mechanism in lung cells [18]. In this study, we determined the mechanism of HCoV-OC43-induced apoptosis in Vero and MRC-5 cells that contributes to viral replication.

Cell death is a common outcome of virus infection and replication [52], and we show that HCoV-OC43 infection decreased cell viability and induced apoptosis in Vero and MRC-5 cells. Considering that the apoptotic cell population is relatively small compared to the decreased cell viability and appears only at late time points (72 h) after virus infection, it is likely that HCoV-OC43 infection majorly induces other types of cell death and apoptosis is an additional form of cell death occurring after several cellular and viral events. It should be noted that large amounts of viral particles are produced at 24 or 48 h after infection without apoptosis. Therefore, cell death such as necrosis, unregulated cell death, or necroptosis seem to be the major forms of cell death. In neuronal cells, HCoV-OC43 induces necroptosis [28,29]. HCoV-OC43 induces caspase activation in neuronal cells, but HCoV-OC43-induced cell death is dependent on Cyclophilin D rather than caspase [28]. Furthermore, the regulated cell death in neuronal cells is dependent on Receptor-Interacting Protein Kinase 1 (RIP1) and Mixed Lineage Kinase Domain-Like (MLKL) [29] which are implicated in necroptosis [23]. As downregulation of RIP1 decreased cell death and increased production of infectious viral particles in neuronal cells, necroptosis seems to limit replication of HCoV-OC43 [29]. Therefore, detailed analysis on the cell death types after HCoV-OC43 infection and their contribution to virus production in Vero and MRC-5 cells remains to be investigated.

The sub-G1 phase was elevated in HCoV-OC43-infected Vero and MRC-5 cells, and S and G2/M phase cells accumulated. Previous studies have shown that cell cycle arrest in S and G2/M phases by virus infection promotes viral replication [53,54]. Coronaviruses such as SADS-CoV, canine CoV, and IBV induce apoptosis via activation of the caspase cascade [16,55,56]. Similarly, HCoV-OC43 infection activated caspase-3 and induced cleavage of one of the main cellular substrates of caspases, PARP, indicating the involvement of caspase in HCoV-OC43-induced apoptosis. Accordingly, the expression of proliferation-related cyclin D1 was decreased. In addition, HCoV-OC43 infection reduced phosphorylation of STAT1 and STAT3 in both Vero and MRC-5 cells. Suppression of STAT1 and STAT3 is associated with the induction of apoptosis [37,38,39] and virus production [40,41,42]. Dephosphorylation or degradation of STAT3 through cellular or viral factors are associated with apoptosis of host cells infected with influenza A virus, hepatitis E virus, and SARS-CoV [41,57,58]. However, the underlying mechanism of STAT1 and STAT3 dephosphorylation in response to HCoV-OC43 infection is to be determined.

As Vero cells cannot produce type I interferons, Vero cells lack competent interferon signaling in response to virus infection. Therefore, we treated Vero cells with IFN-α-2a as an activator of STATs and examined the effect on STAT signaling and virus production. IFN-α-2a increased basal levels of STAT1 and STAT3 phosphorylation as we expected. Furthermore, IFN-α-2a alleviated dephosphorylation of STAT1 and STAT3 upon HCoV-OC43 infection and reduced virus production. Therefore, these results support our suggestion that STAT dephosphorylation is associated with host cell apoptosis and HCoV-OC43 virus replication. We recently found that Vero and Calu-3 cells are susceptible to SARS-CoV-2; however, the two cell types show differences in signaling after infection with respect to STAT activation and apoptosis [59]. In Vero cells, STAT1 and STAT3 phosphorylation disappeared after SARS-CoV-2 infection, as we showed here for HCoV-OC43-infected Vero and MRC-5 cells. In contrast, SARS-CoV-2-infection induced persistent STAT1 and STAT3 phosphorylation in Calu-3 cells. Consistent with the activation status of STATs, apoptotic signals were detected in Vero cells but not in Calu-3 cells after SARS-CoV-2-infection, and higher virus production was found in Vero cells. Since Calu-3 cells are not susceptible to HCoV-OC43, we cannot determine whether this differential signaling depends on virus type or cell type.

z-VAD-FMK, a pan-caspase inhibitor, suppresses the inhibition of virus-induced apoptosis and viral replication in Sindbis virus (SV)- and SADS-CoV-infected cells [16,60]. Pretreatment with z-VAD-FMK significantly decreased the levels of cleaved caspase-3 and cleaved PARP protein in HCoV-OC43-infected Vero and MRC-5 cells and reduced viral production, as determined by HCoV-OC43 N and S protein expression and by plaque assays. These data suggest that activation of caspase-3 is required for HCoV-OC43-mediated apoptosis and viral replication in Vero and MRC-5 cells. However, the effect of z-VAD-FMK on virus replication was marginal compared to the total virus titers even though it was significant. This can be partly explained from the limited efficacy of the inhibitor. We pretreated the cells with z-VAD-FMK for only 1 h, washed, and evaluated the effect on virus replication at 72 h after infection. Furthermore, the viral titers at 72 h represent accumulated ones continuously produced for 72 h after infection in the presence or absence of the caspase inhibitor. It is also necessary to consider that the caspase-3 activation and apoptosis-associated events occurred only at the later times. Collectively, our results suggest that HCoV-OC43 infection induced suppression of STAT signaling and apoptosis to promote viral replication in Vero and MRC-5 cells at least in part.

## 5. Conclusions

HCoV-OC43-mediated dephosphorylation of STAT1 and STAT3 and induction of apoptosis may serve as a mechanism to promote the dissemination of viral progeny in Vero and MRC-5 cells. Viral replication and spread could be limited by treatment with caspase inhibitors, affording the host cell some protection.

## Data Availability

All data needed to evaluate the conclusions in this manuscript have been included. Additional data may be requested from the authors.

## Supplementary Materials

The following are available online at https://www.mdpi.com/article/10.3390/v13112199/s1, Figure S1: Multiplication of HCoV-OC43 in Vero cells, Figure S2: (Corresponding to Figure 2A,B) HCoV-OC43 infection triggers apoptosis in Vero and MRC-5 cells, Figure S3: Effect of HCoV-OC43 infection at high MOI on apoptosis in Vero and MRC-5 cells, Figure S4: (Corresponding to Figure 3) Analysis of relative band densities from western blotting, Figure S5: (Corresponding to Figure 4) Analysis of relative band densities from western blotting, Figure S6: STAT1 and STAT3 phosphorylation at early HCoV-OC43 infection in Vero cells, Figure S7: (Corresponding to Figure 6A) Analysis of relative band densities from western blotting.

## Abbreviations

CCK-8	Cell-counting kit-8
CoVs	Coronaviruses
DMEM	Dulbecco’s modified Eagle’s medium
DMSO	Dimethyl Sulfoxide
EMEM	Eagle’s Minimum Essential Medium
FACS	Fluorescence-activated cell sorting
FBS	Fetal bovine serum
HCoVs	Human coronaviruses
HCoV-OC43	Human coronavirus OC43
HRP	Horseradish peroxidase
IFN	Interferon
IκB	Inhibitory kappa B
MERS-CoV	Middle East respiratory syndrome coronavirus
MOI	Multiplicity of infection
N	Nucleocapsid
NF-κB	Nuclear factor kappa-light-chain-enhancer of activated B cells
PARP	Poly-ADP ribose polymerase
PBS	Phosphate-buffered saline
PCR	Polymerase chain reaction
PI	Propidium iodide
RT-qPCR	Reverse transcriptase-quantitative PCR
S	Spike
SARS-CoV	Severe acute respiratory syndrome coronavirus
STAT1	Signal transducer and activator of transcription 1
STAT3	Signal transducer and activator of transcription 3
