# Supplementary material for: Apoptosis Enhances the Replication of Human Coronavirus OC43
Source: Viruses. 2021 Nov 1;13(11):2199. doi: 10.3390/v13112199 (PMC8619903; doi:10.3390/v13112199)
Supplement: Supplementary file 1 [file viruses-13-02199-s001.zip › viruses-1450826-supplementary.pdf]

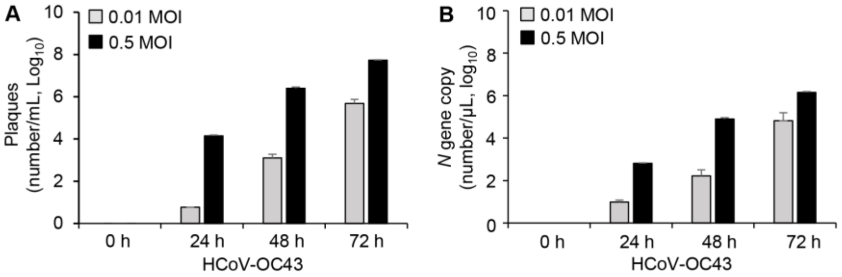

Figure S1. Multiplication of HCoV-OC43 in Vero cells. Vero (2 ×10[5] cells/well) were infected with HCoV-OC43 in 2 ml of DMEM containing 10% FBS at an MOI of 0.01 or 0.5 for 1 h (n=3). At the indicated times after infection, supernatants were collected and analyzed. **(A)** Virus titers in the supernatants were determined by plaque assays. **(B)** Viral replication was quantified by RT-qPCR analysis for the HCoV-OC43 nucleocapsid (*N*) gene in the supernatants. Copy numbers of the *N* gene in 1 μl of the cDNA samples were calculated using a standard curve obtained with cDNA of the *N* gene.

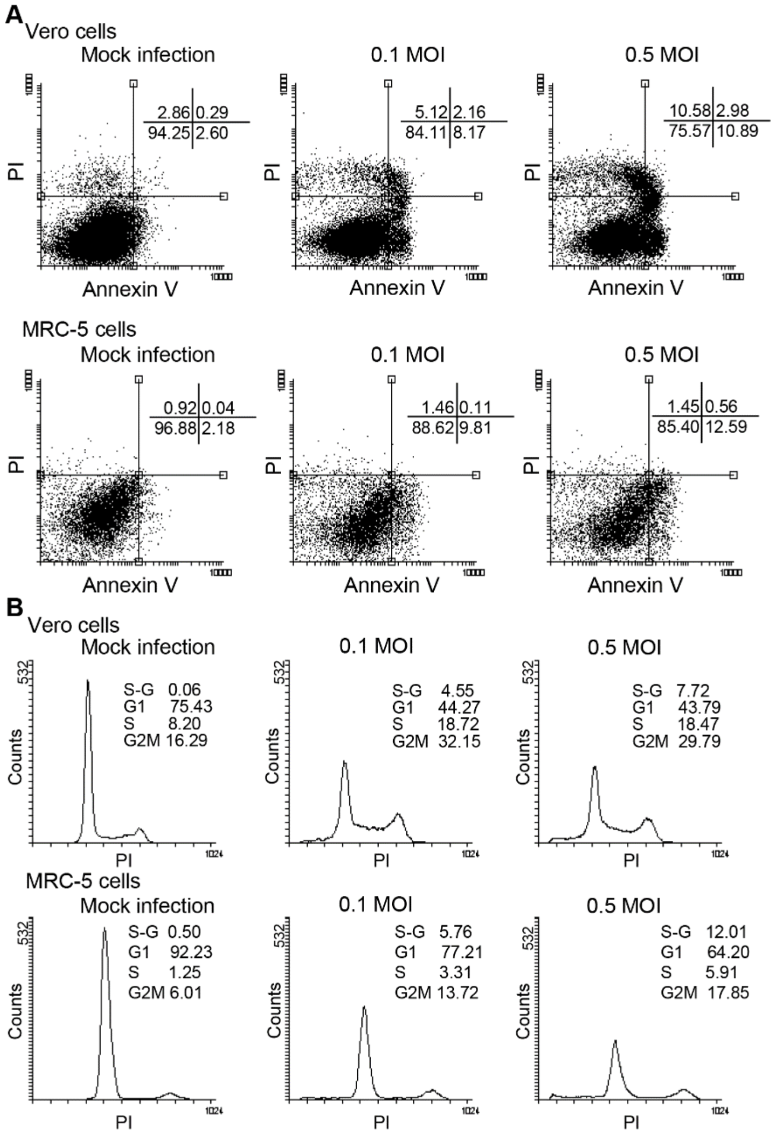

Figure S2. (Corresponding to Figure 2A and 2B) HCoV-OC43 infection triggers apoptosis in Vero and MRC-5 cells. Vero and MRC-5 cells were mock-infected with PBS or infected with HCoV-OC43 in PBS at an MOI of 0.1 or 0.5 for 1 h (n=3). The medium was replaced with DMEM containing 2% FBS or EMEM containing 2% FBS for Vero and MRC-5 cells, respectively. (**A**) Cells harvested at 72 h post-infection were stained with annexin V and PI and subjected to FACS analysis. (**B**) Cells were stained with PI at 72 h post-infection and the distribution of cells at various phases of the cell cycle was determined by flow cytometry. These results are representative of three independent experiments.

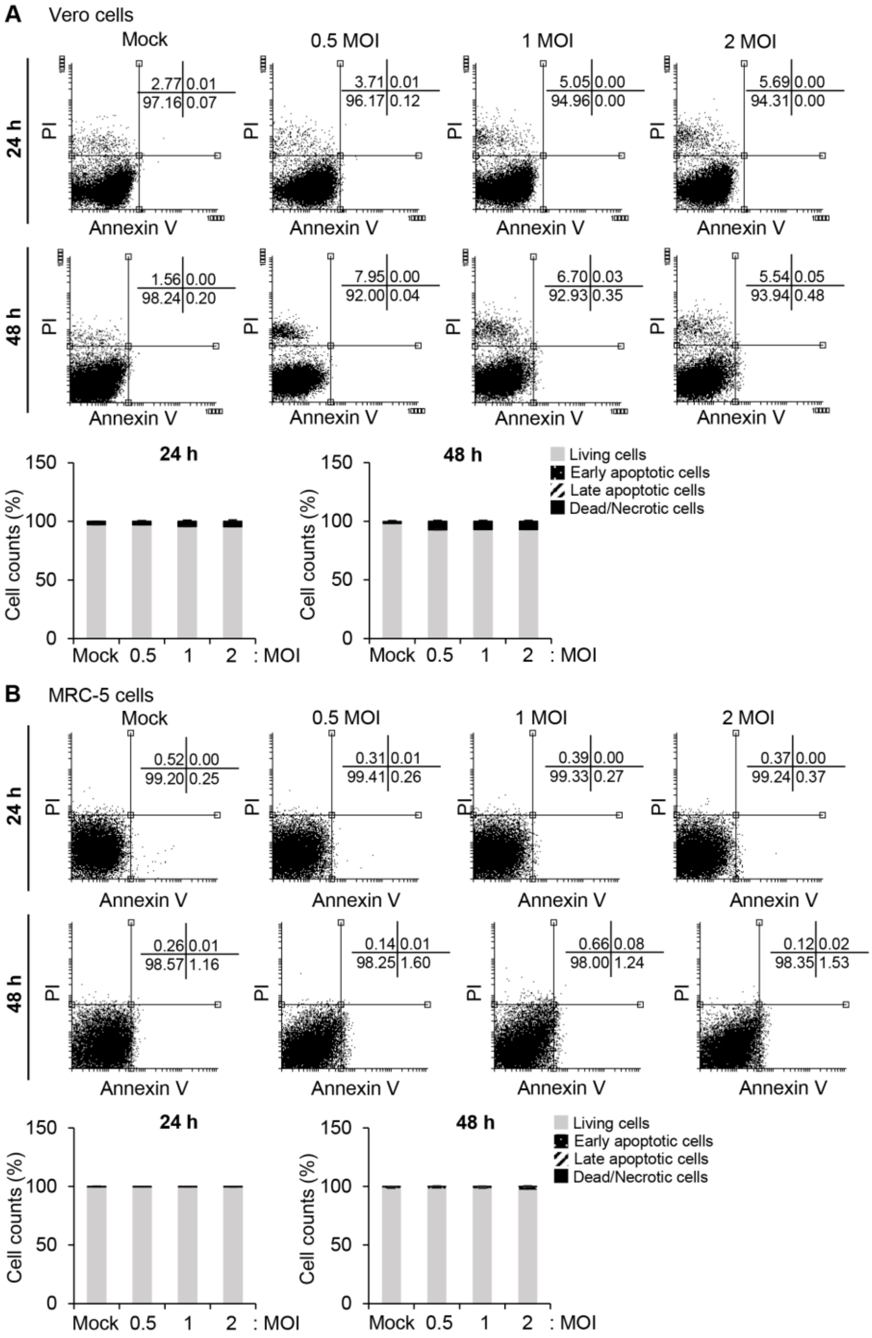

Figure S3. Effect of HCoV-OC43 infection at high MOI on apoptosis in Vero and MRC-5 cells. Vero (**A**) and MRC-5 (**B**) cells were mock-infected with PBS or infected with HCoV-OC43 in PBS at an MOI of 0.5, 1 or 2 for 1 h (n=3). The medium was replaced with DMEM containing 2% FBS or EMEM containing 2% FBS for Vero and MRC-5 cells, respectively. Cells harvested at 24 h and 48 h post-infection were stained with annexin V and PI and subjected to FACS analysis. The bar graphs show HCoV-OC43-induced apoptosis rates and percentage of cells.

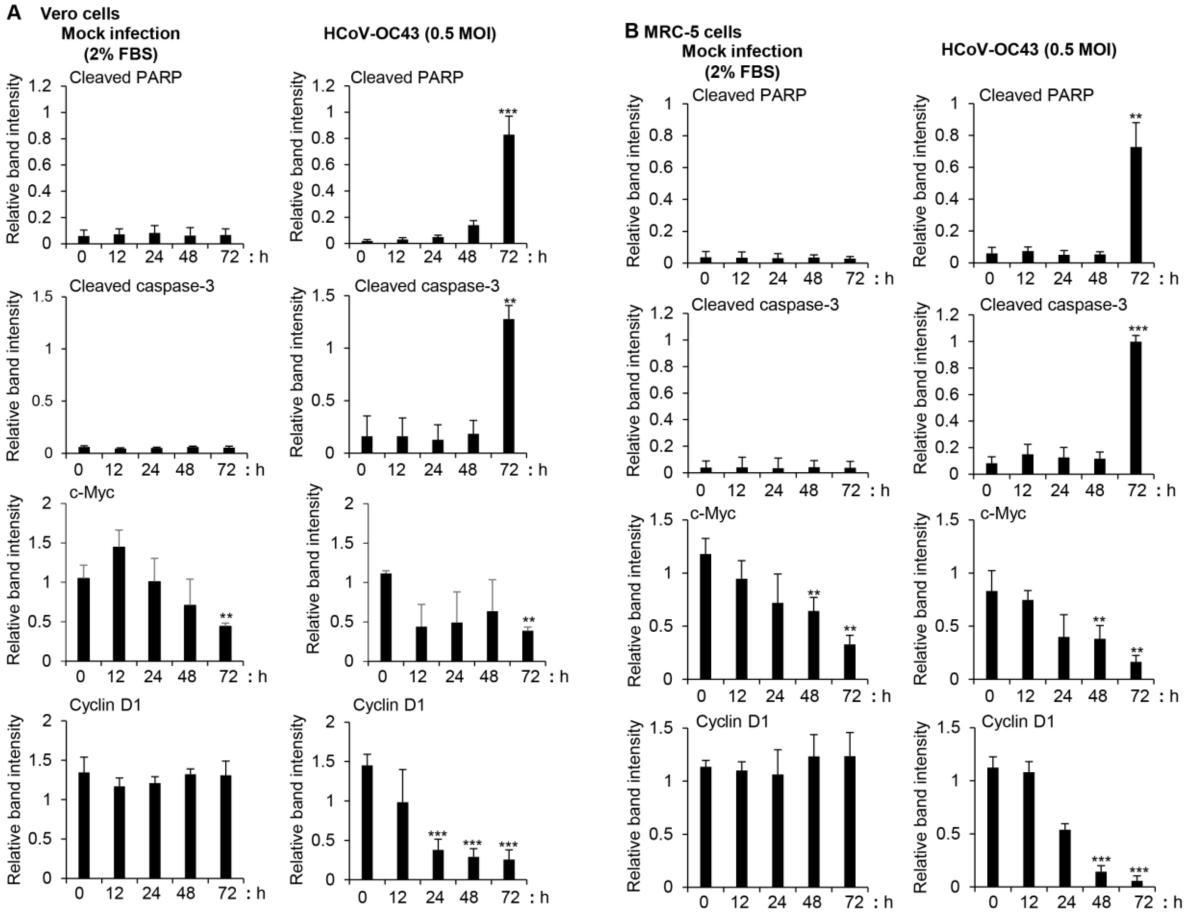

Figure S4. (Corresponding to Figure 3) Analysis of relative band densities from western blotting. (**A** and **B**) Vero (**A**) and MRC-5 (**B**) cells were infected with 0.5 MOI of HCoV-OC43 or without virus infection for the indicated times. After 1 h of incubation, the medium was replenished with DMEM or EMEM containing 2% FBS for Vero and MRC-5, respectively. After the indicated times of incubation, culture medium was removed and cell lysates were prepared, and Western blot analysis was performed to detect PARP, cleaved caspase-3, c-Myc, and cyclin D1. β-actin was used as the control. The band intensities were measured and normalized by β-actin amounts. The relative band intensities are shown on the graph. These results are obtained from three independent experiments. *$p < 0.05$, **$p < 0.01$, ***$p < 0.001$.

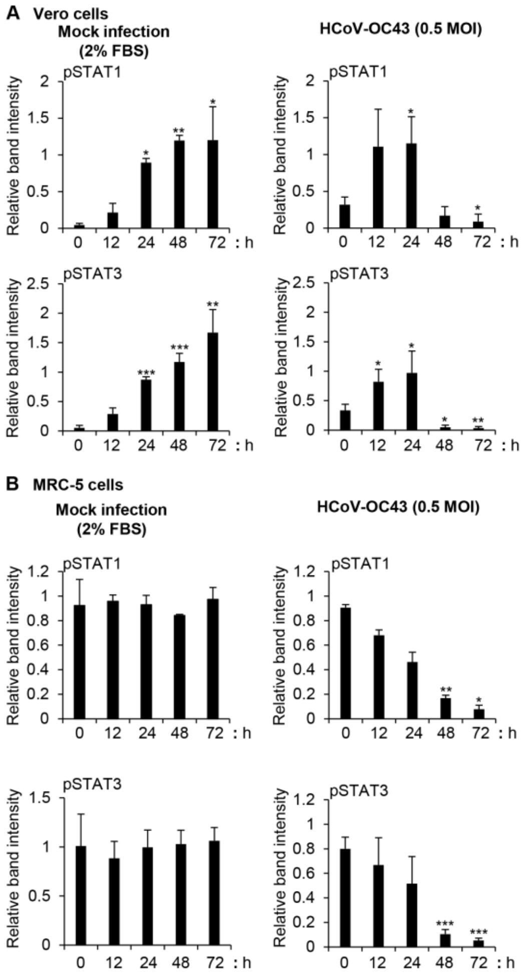

Figure S5. (Corresponding to Figure 4) Analysis of relative band densities from western blotting. (**A** and **B**) Vero (**A**) and MRC-5 (**B**) cells were infected with 0.5 MOI of HCoV-OC43 or without virus infection. After 1 h of incubation, the medium was replenished with DMEM or EMEM containing 2% FBS for Vero and MRC-5, respectively. After the indicated times of incubation, cell lysates were prepared, and Western blot analysis was performed to detect pSTAT1, STAT1, pSTAT3, STAT3. β-actin was used as the control. The band intensities were measured and normalized by β-actin amounts. The relative band intensities are shown on the graph. These results are representative of three independent experiments.

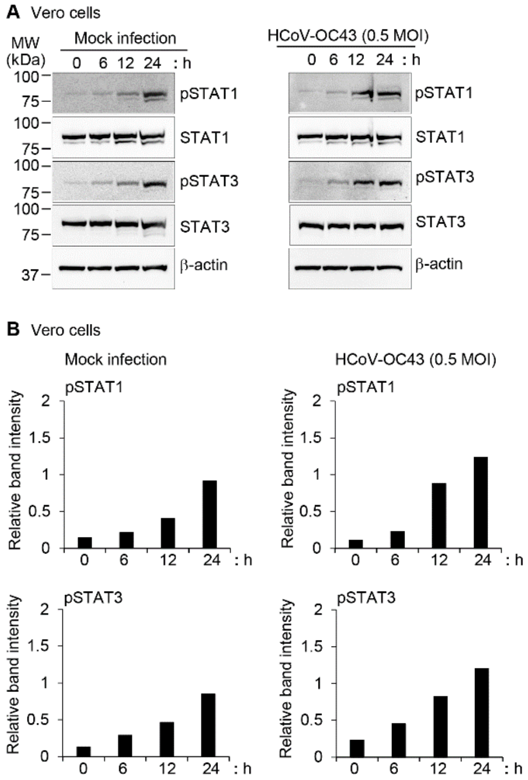

Figure S6. STAT1 and STAT3 phosphorylation at early HCoV-OC43 infection in Vero cells. (**A**) Vero cells were mock-infected with PBS or infected with HCoV-OC43 in PBS at an MOI of 0.5 for 1 h. The medium was replenished with DMEM containing 2% FBS, and cells were grown for the indicated times. At the indicated times after infection, cell lysates were examined by western blotting with antibodies against pSTAT1, STAT1, pSTAT3, STAT3. β-actin antibody was used to verify equal protein loading. (**B**) The band intensities were measured and normalized by β-actin amounts.

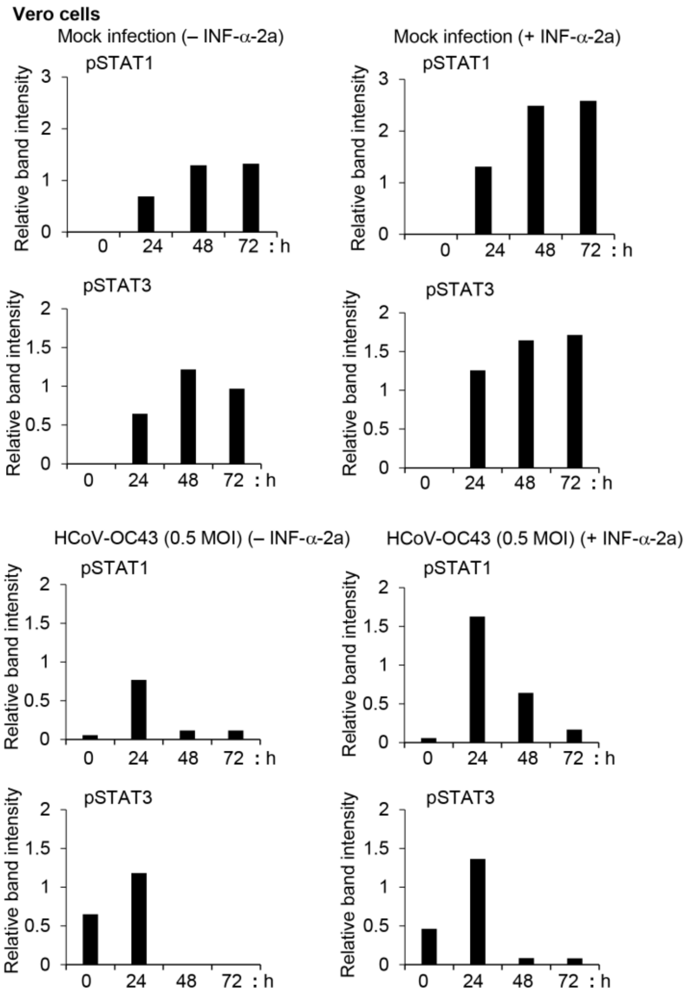

Figure S7. (Corresponding to Figure 6A) Analysis of relative band densities from western blotting. Vero cells were infected with 0.5 MOI of HCoV-OC43 or without virus infection. After 1 h of incubation, the medium was replenished with DMEM containing 2% FBS and 1,000 IU/ml of IFN-α-2a. After the indicated times of incubation, cell lysates were prepared, and western blot analysis was performed to detect pSTAT1, STAT1, pSTAT3, STAT3. β-actin was used as the control. The band intensities were measured and normalized by β-actin amounts. The relative band intensities are shown on the graph.
